# Three-minute SPECT/CT is sufficient for the assessment of bone metastasis as add-on to planar bone scintigraphy: prospective head-to-head comparison to 11-min SPECT/CT

**DOI:** 10.1186/s13550-016-0252-1

**Published:** 2017-01-05

**Authors:** Helle D. Zacho, José A. Biurrun Manresa, Ramune Aleksyniene, June A. Ejlersen, Joan Fledelius, Henrik Bertelsen, Lars J. Petersen

**Affiliations:** 1 Department of Nuclear Medicine and Clinical Cancer Research Center, Aalborg University Hospital, Aalborg, Denmark; 2 Department of Nuclear Medicine, Aalborg University Hospital, Hobrovej 18-22, Postboks 365, 9100 Aalborg, Denmark; 3 Department of Health Science and Technology, Center for Neuroplasticity and Pain, Aalborg University, Aalborg, Denmark; 4 Centro de Investigaciones y Transferencia de Entre Ríos, CONICET-UNER, Entre Ríos, Argentina; 5 Dept. of Nuclear Medicine, Regional Hospital West Jutland, Herning, Denmark

**Keywords:** Bone scintigraphy, SPECT/CT, Fast acquisition, Bone metastases, Agreement

## Abstract

**Background:**

The aim of this study is to assess whether ultra-fast acquisition SPECT/CT (UF-SPECT/CT) can replace standard SPECT/CT (std-SPECT/CT) as “add-on” to whole-body bone scintigraphy (WB-BS) for the investigation of bone metastases.

Consecutive cancer patients referred for WB-BS who underwent SPECT/CT in addition to WB-BS were included. Std-SPECT, UF-SPECT, and low-dose CT were performed (std-SPECT: matrix 128 × 128, zoom factor 1, 20 s/view, 32 views; UF-SPECT: identical parameters except for 10 s/view and 16 views, reducing the acquisition time from 11 to 3 min). A consensus diagnosis was reached by two observers for each set of images (WB-BS + standard SPECT/CT or WB-BS + UF-SPECT/CT) using a three-category evaluation scale: M0: no bone metastases; M1: bone metastases; and Me: equivocal findings.

**Results:**

Among the 104 included patients, most presented with prostate cancer (*n* = 71) or breast cancer (*n* = 28). Using WB-BS + std-SPECT/CT, 71 (68%) patients were classified as M0, 19 (18%) as M1, and 14 (14%) as Me. Excellent agreement was observed between WB-BS + std-SPECT/CT and WB-BS + UF-SPECT/CT using the three-category scale: kappa = 0.91 (95% CI 0.84–0.97). No difference in observer agreement between cancer types was detected. SPECT/CT provided a definitive classification in 90 of 104 cases in which WB-BS was not entirely diagnostic.

**Conclusions:**

To investigate potential bone metastases, UF-SPECT/CT can be conducted as add-on to WB-BS to notably reduce the SPECT acquisition time without compromising diagnostic confidence.

## Background

Whole-body bone scintigraphy (WB-BS) is commonly used to assess the involvement of the skeleton in various cancers, such as prostate and breast cancer [1]. In prostate cancer, WB-BS is the recommended investigation in newly diagnosed patients, in patients with recurrent disease and in patients receiving a treatment response evaluation [2, 3]. The reported sensitivity of WB-BS in diagnosing bone metastases is high, but its specificity is moderate [4, 5].

Since the introduction of WB-BS, technical progress in molecular imaging has emerged in the form of tomographic imaging combined with anatomical mapping, as observed in single-photon emission computed tomography (SPECT) combined with computed tomography (CT) (SPECT/CT). In the last decade, SPECT/CT has become widely available at nuclear medicine clinics in Europe and in the USA [6]. Several studies have reported improved diagnostic confidence when using SPECT/CT as an “add-on” to WB-BS in cases of equivocal or suspicious lesions, particularly due to the increased specificity of SPECT/CT [4, 7–10]. In breast cancer, SPECT/CT may also improve diagnostic sensitivity [4]. Nevertheless, the addition of SPECT/CT to WB-BS is rather time-consuming and may prolong the examination time by 15–20 min for each bed position needed [4, 7]. A long acquisition time may be a challenge in terms of patient capacity and patient compliance; many patients with pain may find it difficult to lie still for 30–40 min for two-bed SPECT/CT. A short acquisition time is technically attainable; however, the acquisition time should not be reduced at the cost of compromised diagnostic confidence.

The aim of the present prospective study was to investigate whether ultra-fast acquisition SPECT/CT can replace standard SPECT/CT without the compromising diagnostic value.

## Methods

### Patients

One hundred and four patients of 503 patients referred for BS at our department were enrolled in this prospective study from July 2014 to January 2015. The inclusion criteria were as follows: (1) a diagnosis of cancer; (2) referral as part of a routine clinical bone scintigraphy examination due to the suspicion of bone metastases; (3) performance of SPECT/CT in accordance with institutional practice as required by the nuclear medicine physician in charge as an add-on to WB-BS in cases of (a) equivocal lesions on WB-BS or (b) patient complaints of localized, cancer-suspicious pain despite normal WB-BS findings; and (4) receipt of a signed consent form.

All patients filled out a standard questionnaire regarding bone-related pain and known benign bone conditions that might impact the diagnostic classification of the bone scintigraphy results, such as recent trauma, osteoarthritis, or recent surgery to bones or joints.

### Bone scintigraphy

#### WB-BS

Standard WB-BS was performed in accordance with the European Society of Nuclear Medicine guidelines [11]. Tc-99m-labeled methylene bisphosphonate (750–1000 MBq, 20–27 mCi) was injected 2 to 3 h before image acquisition. The patients were orally hydrated, and they voided their bladders immediately prior to undergoing WB-BS. Symbia dual-head gamma cameras (Symbia T16, Siemens Medical Solutions, Erlangen, Germany) capable of simultaneous anterior and posterior acquisition and equipped with multi-purpose, low-energy, high-resolution collimators were used. The scan speed was 24 cm/min, with alpha blending of 30% [12], a matrix size of 256 × 1024, and a zoom factor of 1. The WB-BS images were evaluated by the physician in charge, who decided on the need for additional SPECT/CT (as well as the number of bed positions).

#### SPECT

Standard SPECT (std-SPECT) was conducted using the following parameters: matrix 128 × 128, zoom factor 1, 20 s per view, 32 views, rotation of the detectors by 180° in a non-circular orbit, and step-and-shoot mode. Ultra-fast acquisition SPECT (UF-SPECT) was conducted using the same parameters as std-SPECT, except for 16 views of 10 s per view; thus, the effective acquisition time was reduced from 11 min (10 min 40 s) for std-SPECT to 3 min (2 min 40 s) for UF-SPECT. The total camera time for SPECT was reduced from 13 min 20 s to 4 min 50 s. Resolution recovery (Flash 3D) iterative reconstruction (four iterations over eight subsets) with scatter correction was used for both types of SPECT. The parameters for UF-SPECT acquisition were chosen based on an exploratory pilot study conducted before initiating the present study.

#### CT

Low-dose CT was performed for attenuation correction and anatomical co-registration using the following parameters: 25 mA, 130 peak keV, scan time 13.55 s, 30 mAs, and slice thickness 0.6 mm. Only one CT scan was performed and used for both std-SPECT and UF-SPECT. Images were fused using the manufacturer’s software (e.soft™ application on the Symbia.net™ Clinical Workflow System, Siemens Healthcare, Erlangen, Germany). Patients were allocated to one of two sequences according to their date of birth: (1) std-SPECT, low-dose CT followed by UF-SPECT, or (2) UF-SPECT, low-dose CT followed by std-SPECT. WB-BS and std-SPECT/CT were used for the clinical report (i.e., the results of UF-SPECT/CT were not used for clinical purposes).

### Observers and procedure for image assessment

Four board-certified nuclear medicine physicians with 7–10 years of experience from two departments of nuclear medicine (Aalborg and Herning) evaluated the UF-SPECT/CT and re-evaluated the std-SPECT/CT. All of the observers were experienced with std-SPECT/CT. The observers had access to the clinical data provided by the referring physician and to the bone-related questionnaire responses. They had no access to the previous imaging data or to the clinical report of the WB-BS + std-SPECT/CT findings. The observers from Herning had approximately 1 year of clinical experience with UF-SPECT/CT in selected patients. In contrast, the observers from Aalborg had no experience with UF-SPECT/CT.

The images for each patient were examined by two observers, one from each department. The WB-BS + std-SPECT/CT or WB-BS + UF-SPECT/CT images were retrieved to the workstation. The images were loaded in allocated workflows for either std-SPECT/CT or UF-SPECT/CT; this method allowed the observers to individually adjust the brightness and contrast settings of the images and fusion of the images if desired. Consequently, the individual observers were not blinded to the type of SPECT/CT.

For the individual patient, the two acquisition types (std-SPECT/CT and UF-SPECT/CT) were evaluated on separate days and the patients were randomly assigned to either std-SPECT/CT (including WB-BS) or UF-SPECT/CT (including WB-BS) at the first evaluation day. In order to prevent the observers from recalling the individual patient, the interval between reading days was at least 4 weeks. After individual assessment of the images, consensus was reached between the two observers in cases of disagreement; there was no need for a third party arbitrator in any case. If the individual observer considered the image quality to be inadequate, they were given the opportunity to deem the investigation or part of the investigation as “not of diagnostic quality”.

### Diagnostic classification

The observers classified each std-SPECT/CT and UF-SPECT/CT result separately into one of three categories: (1) normal findings or findings considered to represent benign conditions (M0), (2) equivocal findings in which changes were observed on std-SPECT/CT or UF-SPECT/CT but could not be categorized as benign or malignant (Me), or (3) findings of bone metastases (M1). Furthermore, the observers reported the anatomical localization of the bone metastases or equivocal lesions. The skeleton was divided into seven anatomical areas: (a) head; (b) thorax, including the sternum; (c) cervical and thoracic spine; (d) lumbar spine; (e) pelvis, including the sacrum; (f) upper extremities, including the scapulae; and (g) lower extremities. For each anatomical area, the number of equivocal or malignant lesions was recorded as “0,” “1,” or “>1”. This classification was used to determine whether agreement between the acquisition methods was based on the same lesions.

### Statistics

Agreement was assessed using Cohen’s kappa (*κ*), which is calculated as the ratio of the overall percent agreement corrected for chance to the maximum possible percent agreement corrected for chance. We used the linear weighted *κ*, and disagreements were ranked according to the distance between the categories (e.g., a disagreement from “0” to “>1” was assigned greater weight than a disagreement from “0” to “1”). Furthermore, maximum achievable kappa values (*κ*_max_) given the marginal distributions are reported, to determine how much of the marginally permitted agreement is present between raters [13]. The magnitude of agreement was classified as slight (kappa 0–0.20), fair (0.21–0.40), moderate (0.41–0.60), substantial (0.61–0.80), or almost perfect agreement (0.80–1.0) in accordance with standardized criteria [14]. Additionally, the Bhapkar test was performed to assess marginal homogeneity, i.e., to test if raters have different propensities to use each rating category. The null hypothesis for Bhapkar’s test is that marginal probabilities are homogeneous; *χ* ^2^(degrees of freedom) and *p* values are reported. All results are reported with 95% confidence intervals (95% CI), and the significance level was set to 0.05.

### Ethics

This study complied with the Helsinki II Declaration. The study protocol was assessed by the North Denmark Region Committee on Health Research Ethics and the Danish Data Protection Agency. All patients provided signed informed consent to participate.

## Results

### Patients

A total of 104 patients (30 women and 74 men, aged 69 ± 10 years) were recruited for this study. The majority of the included patients had prostate cancer (*n* = 71, 68%) or breast cancer (*n* = 28, 27%), and five (5%) patients suffered from other cancer types or several cancer types. A total of 48 patients (all with prostate cancer) were referred for staging, 26 patients were referred due to bone-related pain, 18 patients were referred because biomarker (PSA or alkaline phosphate) findings indicated bone metastases, and 12 patients were referred for various other reasons.

### Image quality and prevalence of bone metastasis

**Fig. 1:**
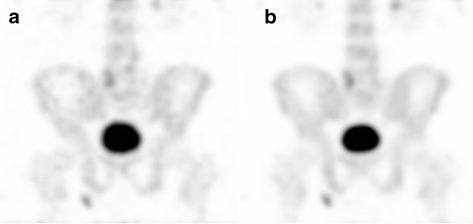
Illustrative examples of the M0 category (no bone metastases); in this example, tracer uptake was increased in the right side of L5 corresponding to benign changes seen on low-dose CT. **a** Anterior view of a maximum intensity projection (MIP) from ultra-fast acquisition SPECT. **b** Anterior view of a MIP from standard acquisition SPECT

**Fig. 2:**
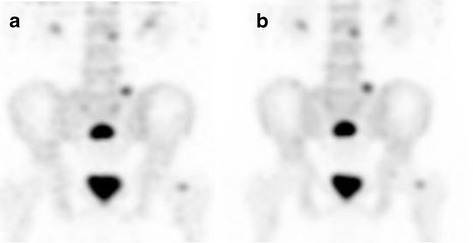
Illustrative examples of the M1 category (bone metastases); in this example, tracer uptake was increased in the os sacrum and proximal right femur corresponding to osteosclerotic lesions on low-dose CT. Additionally, tracer uptake was increased in the left side of L3 and L5 corresponding to benign changes seen on low-dose CT. **a** Posterior view of a maximum intensity projection (MIP) from ultra-fast acquisition SPECT. **b** Posterior view of a MIP from standard acquisition SPECT

The technical quality of std-SPECT, UF-SPECT, and low-dose CT was classified as sufficient for diagnostic purposes by all readers for all patients. Examples of patients with malignant and benign findings are shown in Figs. 1 and 2. Std-SPECT/CT showed bone metastases (M1) in 19 (18%) patients, no bone metastases (M0) in 71 (68%) patients, and equivocal results for bone metastasis in the remaining 14 (14%) patients.

### Agreement between std-SPECT/CT and UF-SPECT/CT

**Fig. 3:**
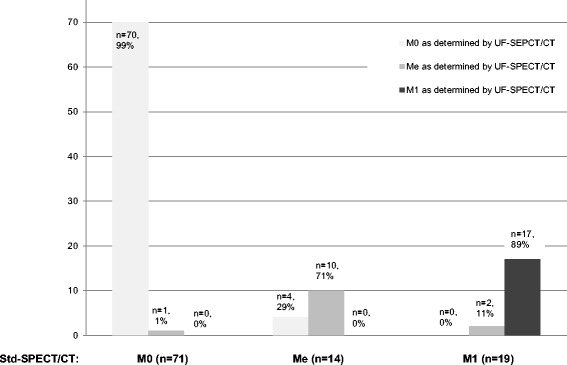
Consensus diagnosis by two physicians using std-SPECT/CT according to a three-category scale: M0: no bone metastasis; Me: changes observed on BS that could not be categorized as benign or malignant; and M1: bone metastases. For each category, the consensus diagnosis for UF-SPECT/CT is shown

Uniform agreement between the consensus diagnosis (M1, M0, and Me) using UF-SPECT/CT and the consensus diagnosis using std-SPECT/CT was observed in 97 of 104 (93%) patients (Fig. 3). The linear weighted Cohen’s *κ* was excellent (*κ* = 0.91, 95% CI 0.84–0.97; *κ*_max_ = 0.93; *χ* ^2^(2) = 3.94, *p* = 0.139). Among the seven patients with discrepancy between UF and SD SPECT, four out of seven suspicious/equivocal lesions were in the pelvic region and three out of seven lesions were in the thorax region. Thus, no apparent issue with attenuation mass in the abdomen or pelvis was observed. The agreement between std-SPECT/CT and UF-SPECT/CT was similar between prostate cancer patients (*κ* = 0.90, 95% CI 0.82–0.99; *κ*_max_ = 0.94; *χ* ^2^(2) = 2.41, *p* = 0.299) and breast cancer patients (*κ* = 0.90, 95% CI 0.77–1; *κ*_max_ = 0.90 *χ* ^2^(2) = 2.18, *p* = 0.336). Due to the sample size, no such calculation was performed for other types of cancer.

Consensus reading was necessary in 12 of 104 patients (12%) for UF-SPECT/CT, compared to 15 of 104 patients (14%) for std-SPECT/CT. In seven patients, consensus reading was necessary for both UF-SPECT/CT and std-SPECT/CT.

In 102 of 104 patients (98%), there was agreement on the numbers and localization of the regions of suspicious/malignant lesions using the two SPECT/CT acquisition methods. In one patient, there was disagreement in the number of regions involved, two regions were suspicious according to UF-SPECT/CT, but only one of the regions was considered suspicious by std-SPECT/CT. Finally, the observers considered suspicious lesions/bone metastases in completely different regions in one patient on both std-SPECT/CT and UF-SPECT.

## Discussion

Timely and accurate diagnosis of bone metastases is critical to ensure that cancer patients receive the appropriate treatment. WB-BS has been the cornerstone in such situations for many years. The use of additional SPECT/CT for evaluating suspicious or equivocal lesions observed on WB-BS has been shown to improve diagnostic confidence [4, 7, 8, 10], but the resulting increase in acquisition time is undesirable. In the present study, we investigated the use of UF-SPECT/CT as add-on to WB-BS for confirming or excluding the presence of bone metastases in cancer patients, and we found excellent agreement between UF-SPECT/CT and std-SPECT/CT for this task. The conclusion of this study is that UF-SPECT/CT is suitable for clinical practice.

This study included patients with different types of cancer, although the majority of the patients presented with prostate or breast cancer. Bone metastases from prostate and breast cancer differ in terms of morphology: the metastases from prostate cancer are largely osteosclerotic, and those from breast cancer are a mixture of osteolytic and osteosclerotic. For this reason, the agreement between UF-SPECT/CT and std-SPECT/CT was separately assessed for prostate cancer and breast cancer. There were no clinically relevant differences in the diagnostic classification between UF-SPECT/CT and std-SPECT/CT among prostate and breast cancer patients. This result demonstrates the applicability of UF-SPECT/CT independent of the presumed histology of the lesion.

A three-category scale of M1, M0, and Me was chosen to reflect the clinical reality, in which WB-BS, with or without SPECT/CT, cannot distinguish whether a lesion is malignant or benign with certainty in all patients. Large studies performing WB-BS on prostate cancer patients have revealed that 16–26% of these patients show equivocal WB-BS findings [15, 16]. Previous studies performing SPECT/CT to evaluate bone metastasis have inconsistently reported the frequency of equivocal scan results. We have previously shown that there is a need for an equivocal category in the reading of bone scans to avoid diagnostic misclassification [17]; we encourage the inclusion of this category for the classification of SPECT/CT images of the bone, as well. The number of categories increases the options available to observers and thereby decreases interobserver agreement. Even though the findings were categorized into three classes rather than two (M1 or M0), the interobserver agreement was very satisfactory. In the present study, 14% of the patients showed equivocal std-SPECT/CT findings. This percentage might appear rather high, but in the present study, SPECT/CT was only conducted as add-on on patients for whom WB-BS was not entirely diagnostic. Under these conditions, SPECT/CT provided a definitive classification in 90 of 104 cases.

The excellent agreement between the two acquisition types in the present study was emphasized by the identification of suspicious/malignant lesions in identical regions and numbers by the observers in the vast majority of the patients.

The observers were not blinded to the type of SPECT/CT. The UF-SPECT/CT images were significantly less smooth than the std-SPECT/CT images. It could be argued that the observers’ knowledge of the modality in which they were interpreting was a weakness of the present study. To hinder observers from recalling specific patients at least 4 weeks were interposed between the evaluation of std-SPECT/CT and the evaluation of UF-SPECT/CT. Despite the grainy appearance of the UF-SPECT/CT images, all images were considered by the observers to be of diagnostic quality and to be supportive of an inconclusive WB-BS result. An explanation for our findings could be that the UF-SPECT/CT—in the majority of cases—was used for anatomical co-registration of suspicious lesions seen on the WB-BS, and for this purpose, less smooth images appear to be sufficient.

Recently, there has been growing interest in the use of checklists in diagnostic test accuracy studies, such as STARD to describe the competencies of the observers, as their interpretation directly affects the performance of the diagnostic test [18–20]. We used observers from two different departments of nuclear medicine, one institution that had slight experience with UF-SPECT/CT and one without such experience. Evaluation of new modalities by several observers from different departments is an important factor to consider when determining whether a new method is widely applicable. The excellent agreement in the present study indicates that UF-SPECT/CT can be introduced in departments familiar with std-SPECT/CT without previous training in UF-SPECT/CT.

For the present study, identical reconstruction algorithms using iterative reconstruction with resolution recovery were applied to std-SPECT/CT and UF-SPECT/CT. Aldridge et al. have investigated the use of different reconstruction algorithms in parathyroid and bone SPECT/CT. They found that the use of 3D resolution recovery for image reconstruction resulted in a 50% decrease in acquisition time without reducing image quality. The use of a 75% reduction in acquisition time was also investigated, and no reduction in image quality was observed compared to filtered back projection [21]. Our results are supported by Stansfield et al. who investigated image quality in half-time SPECT in pediatric patients undergoing BS. They found negligible differences in image quality, noise reduction, and image sharpness between full- and half-time SPECT [22]. Thientunyakit et al. investigated the use of half-time SPECT (no CT) compared to multiplanar imaging of the pelvis in patients with significant bladder artifacts on WB-BS. They concluded that the use of half-time SPECT increased the diagnostic confidence compared to multiplanar imaging and that the half-time SPECT images were of diagnostic quality [22]. Reduction of acquisition time has particularly been investigated for myocardial perfusion imaging using SPECT. In general, those studies reported that the use of alternative acquisition or reconstruction algorithms compensated for the loss of image quality resulting from a reduced scan time. No change in reconstruction algorithms was used in the present study, and the image quality was not assessed specifically.

Despite that, SPECT/CT has been widely accessible for more than a decade; studies have hitherto focused on half-time SPECT. In the present study, the std-SPECT was in fact a half-time SPECT compared to SPECT with filtered back projection and the UF-SPECT method used in the present study reduced acquisition time to 25%. Such short acquisition times have not been investigated before, possibly due to concerns regarding reduced image quality. However, none of the UF-SPECT/CT images was deemed unsuitable for diagnosis, even though the short-duration acquisition produced coarser images. On the other hand, the short acquisition time may be considered more comfortable to patients, and consequently, the patients might be less likely to move during the acquisition period.

For the future studies to optimize SPECT acquisition balancing image quality versus time, we await that list mode data becomes available on modern SPECT systems enabling the user to parse the data and create acquisitions with any proportion of counts from the full-time dataset, with no extra scans needed for the patient. Even without list mode data, this can be achieved by acquiring the SPECT as a gated dataset (i.e., with ECG trigger), and reconstruction of only a proportion of the time-bins.

## Conclusions

In conclusion, UF-SPECT/CT can replace std-SPECT/CT as add-on to WB-BS for the evaluation of inconclusive lesions observed on WB-BS regardless of the cancer type. Using UF-SPECT/CT, the SPECT acquisition time can be reduced by 75% without compromising diagnostic confidence.
